# Mutualistic co‐evolution of T3SSs during the establishment of symbiotic relationships between *Vigna radiata *and Bradyrhizobia

**DOI:** 10.1002/mbo3.781

**Published:** 2019-01-09

**Authors:** Pongdet Piromyou, Pongpan Songwattana, Kamonluck Teamtisong, Panlada Tittabutr, Nantakorn Boonkerd, Piyada Alisha Tantasawat, Eric Giraud, Michael Göttfert, Neung Teaumroong

**Affiliations:** ^1^ School of Biotechnology, Institute of Agricultural Technology Suranaree University of Technology Nakhon Ratchasima Thailand; ^2^ The Center for Scientific and Technological Equipment Suranaree University of Technology Nakhon Ratchasima Thailand; ^3^ School of Crop Production Technology, Institute of Agricultural Technology Suranaree University of Technology Nakhon Ratchasima Thailand; ^4^ IRD, Laboratory of Tropical and Mediterranean Symbioses UMR IRD/SupAgro/INRA/UM2/CIRAD, Campus International de Baillarguet Montpellier France; ^5^ Institute of Genetics TU Dresden Dresden Germany

**Keywords:** bradyrhizobial T3SS, mutualistic co‐evolution, *V. radiata*–bradyrhizobia symbiosis

## Abstract

This study supports the idea that the evolution of type III secretion system (T3SS) is one of the factors that controls *Vigna radiata*–bradyrhizobia symbiosis. Based on phylogenetic tree data and gene arrangements, it seems that the T3SSs of the Thai bradyrhizobial strains SUTN9‐2, DOA1, and DOA9 and the Senegalese strain ORS3257 may share the same origin. Therefore, strains SUTN9‐2, DOA1, DOA9, and ORS3257 may have evolved their T3SSs independently from other bradyrhizobia, depending on biological and/or geological events. For functional analyses, the *rhcJ* genes of ORS3257, SUTN9‐2, DOA9, and USDA110 were disrupted. These mutations had cultivar‐specific effects on nodulation properties. The T3SSs of ORS3257 and DOA9 showed negative effects on *V. radiata* nodulation, while the T3SS of SUTN9‐2 showed no effect on *V. radiata* symbiosis. In the roots of *V. radiata* CN72, the expression levels of the *PR1* gene after inoculation with ORS3257 and DOA9 were significantly higher than those after inoculation with ORS3257 *ΩT3SS*, DOA9 *ΩT3SS,* and SUTN9‐2. The T3Es from ORS3257 and DOA9 could trigger *PR1* expression, which ultimately leads to abort nodulation. In contrast, the T3E from SUTN9‐2 reduced *PR1* expression. It seems that the mutualistic relationship between SUTN9‐2 and *V. radiata* may have led to the selection of the most well‐adapted combination of T3SS and symbiotic bradyrhizobial genotype.

## 1 INTRODUCTION

Despite the massive number of number of bacteria in nature, only a few species can form symbiotic nodules with leguminous plants. Rhizobia are one of the best‐studied examples of a plant microbiota and serve as a model for understanding plant–microbe interactions. In many cases, bacterial mutualistic lineages exhibit intimate interactions with their host. Bradyrhizobia can establish symbiotic relationships with several types of leguminous plants. Although Nod factors allow bradyrhizobia to enter the root hairs of leguminous plants, other bacterial substances are required for productive infection and nodule development (Mathis et al., 2005). Among these factors are proteins secreted via the type III secretion systems (T3SSs; Okazaki, Kaneko, Sato, & Saeki, 2013; Tsukui et al., 2013). T3SSs are a kind of specialized apparatus used for protein secretion by many Gram‐negative bacteria. The secreted proteins of bradyrhizobia are designated as type III effector proteins (T3Es; Marie, Broughton, & Deakin, 2001). T3SSs deliver T3Es directly into the extracellular environment or into the cytosol of eukaryotic host cells (Cornelis & Van Gijsegem, 2000; He, Nomura, & Whittam, 2004; Pallen, Chaudhuri, & Henderson, 2003). Previous reports showed that T3SSs may have either positive or negative effects on leguminous plant–rhizobia symbiosis (Freiberg et al., 1997; Hueck, 1998; Okazaki et al., 2010; Piromyou et al., 2015; Skorpil et al., 2005). Moreover, the T3SS of *Sinorhizobium fredii* NGR234 is functional and is involved in the determination of the host range of nodulation (Viprey, Greco, Golinowski, Broughton, & Perret, 1998).

The mung bean (*Vigna radiata*) is cultivated mostly in South, East, and Southeast Asia by smallholder farmers for its edible seeds and sprouts. The domestication of the mung bean was initiated in the northeast and far south of India approximately 4,000–6,000 years ago (Fuller, 2007). The domesticated mung bean is thought to have spread mainly throughout Southeast Asia from India via different routes (Tomooka, Lairungreang, Nakeeraks, Egawa, & Thavarasook, 1992). Bradyrhizobia are commonly found to establish symbiotic interactions with *V. radiata *in Thailand (Piromyou et al., 2017; Yokoyama et al., 2006); in particular, *Bradyrhizobium* sp. SUTN9‐2 can form symbiotic nodules with many of the *V. radiata *cultivars tested. In contrast, several T3Es from bradyrhizobial strains are major negative effectors for *V. radiata* symbiosis (Nguyen, Miwa, Kaneko, Sato, & Okazaki, 2017; Songwattana et al., 2017; Wenzel, Friedrich, Göttfert, & Zehner, 2010). Thus, SUTN9‐2 is a good model for the symbiotic partnership between *V. radiata *and *Bradyrhizobium*. Nevertheless, the genetic basis of how T3SSs are involved in the enhancement and suppression of nodulation in both partners in *V. radiata* bradyrhizobia symbiotic relationships has not been clearly elucidated. Therefore, the current study is an important step toward understanding the functions of T3SSs in mutualistic relationships.

## 2 RESULTS AND DISCUSSIONS

**Figure A1:**
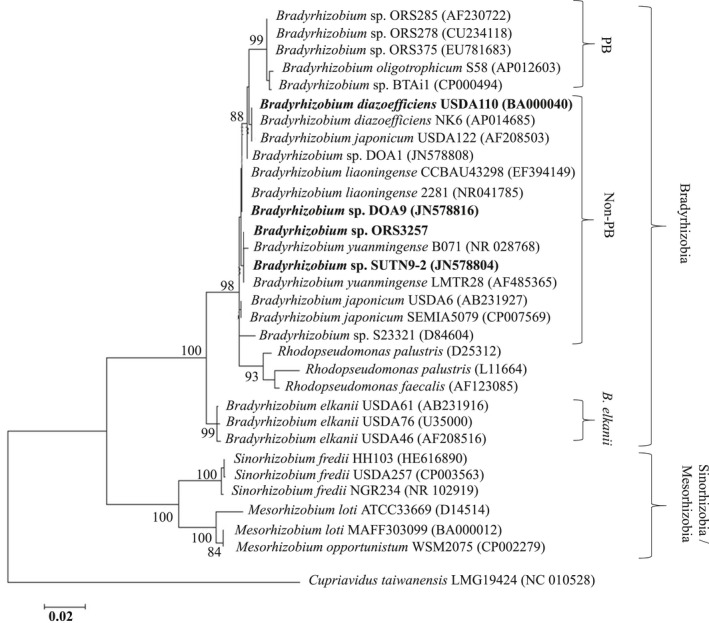
Maximum‐likelihood tree based on combined sequences of 16s rRNA gene, showing classification of bradyrhizobial group and sinorhizobial/mesorhizobial group. Bootstrap values are expressed as percentages of 1,000 replications. The bar represents one estimated substitution per 100 nucleotide positions. Evolutionary distances were computed using the Kimura two‐parameter method and are shown in units representing the number of base substitutions per site

To examine the evolutionary relationships of bradyrhizobial strains, the nucleotide sequences of the 16s rRNA gene from various reference strains of *Bradyrhizobium*, *Sinorhizobium*, *Mesorhizobium,* and *Rhodopseudomonas *species were used to construct a phylogenetic tree. *Cupriavidus taiwanensis *LMG19424 was chosen as the outgroup strain to root the phylogenetic tree (Figure A1). The DNA sequences were generated, and the most closely related sequences were obtained from the NCBI database. The nucleotide sequences were aligned using the ClustalW program, and the phylogenetic trees based on the 16S rRNA and T3SS gene sequences were constructed using the maximum‐likelihood method with PhyML (Guindon & Gascuel, 2003). Based on the 16S rRNA gene sequence similarity, the phylogenetic tree could be divided into two major clusters. Cluster 1 included various groups of *Bradyrhizobium *species, including non‐photosynthetic bradyrhizobia (non‐PB), photosynthetic bradyrhizobia (PB), *B. elkanii *species, and *Rhodopseudomonas *members. The bradyrhizobial strains SUTN9‐2, DOA1, DOA9, ORS3257 and *B. diazoefficiens* USDA110 belonged to the non‐PB cluster. Moreover, strain SUTN9‐2 was closely related to strain ORS3257. Both sinorhizobial and mesorhizobial species were located in major cluster 2 of the phylogenetic tree.

**Figure 1:**
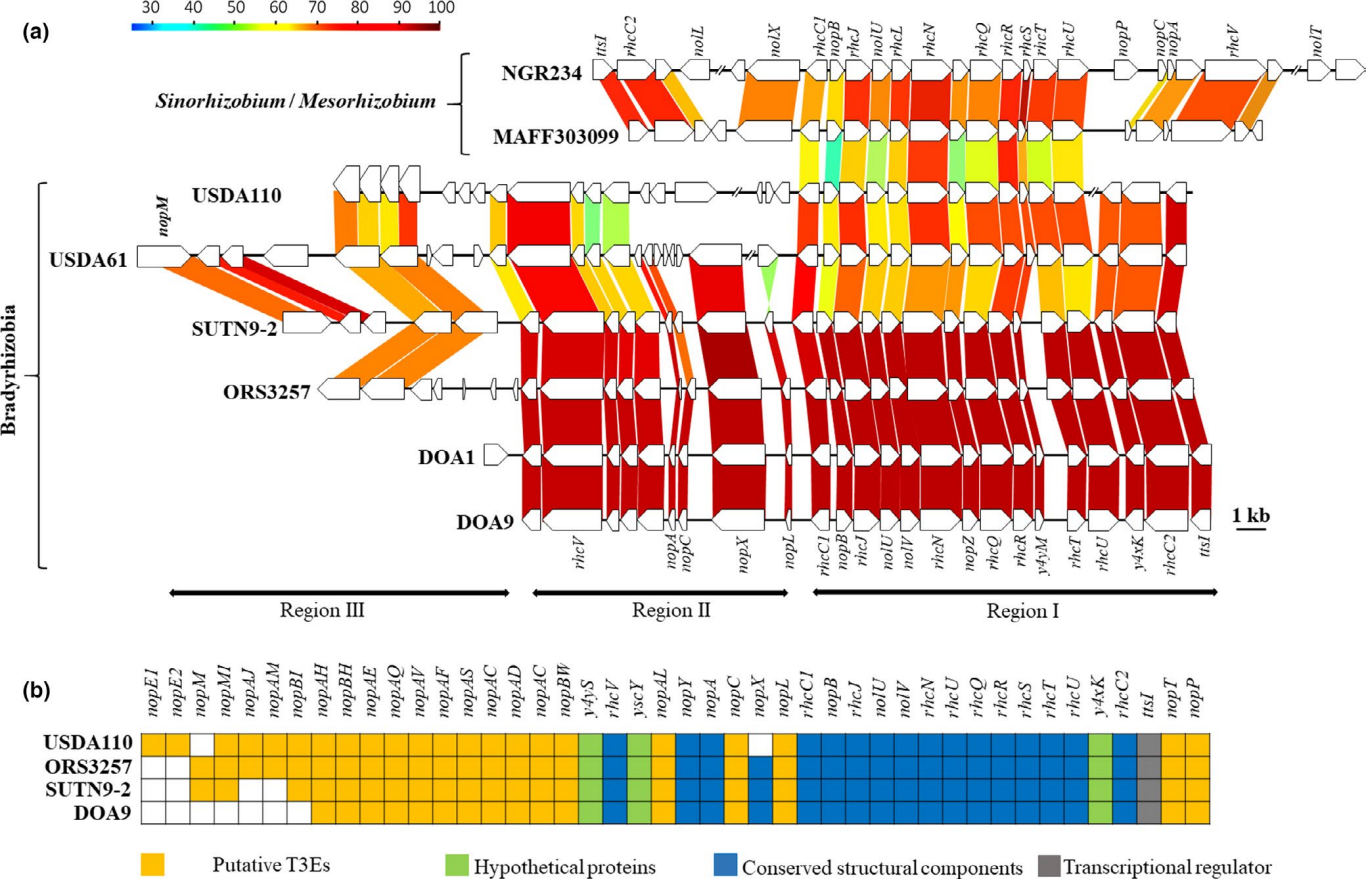
(a) Comparison of the gene organizations of the type III secretion system (T3SS) in the bacterial strains *Sinorhizobium* sp. NGR234, *Mesorhizobium loti* MAFF303099, *Bradyrhizobium diazoefficiens* USDA110, *B. elkanii* USDA61, *Bradyrhizobium* sp. SUTN9‐2, *Bradyrhizobium* sp. ORS3257, *Bradyrhizobium* sp. DOA1, and *Bradyrhizobium* sp. DOA9. The orientations and sizes of the predicted ORFs are depicted by open arrows. Double slash marks represent DNA regions that are not shown. Colored strips represent the conserved gene regions among the compared strains, and the colors indicate the similarity percentage. (b) Distribution of the conserved structural components and some putative T3E families in bradyrhizobia. The structural components and T3E family names are listed across the top with bradyrhizobial strains. Boxes are color‐coded as indicated in the key; white boxes: no detectable homology; yellow boxes: putative T3Es; green boxes: hypothetical proteins; blue boxes: conserved structural components, and gray boxes: transcriptional regulator

**Figure A2:**
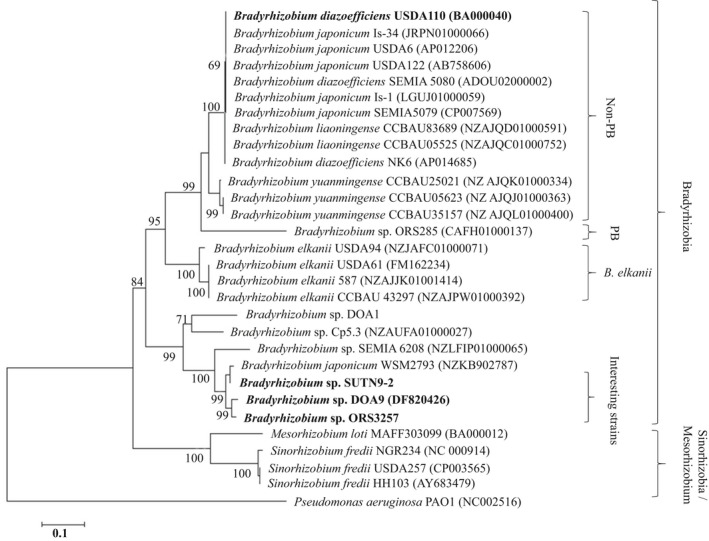
Maximum‐likelihood tree based on combined sequences of *rhcJ* gene, showing classification of bradyrhizobial group and sinorhizobial/mesorhizobial group. Bootstrap values are expressed as percentages of 1,000 replications. The bar represents one estimated substitution per 100 nucleotide positions. Evolutionary distances were computed using the Kimura two‐parameter method and are shown in units representing the number of base substitutions per site

A phylogenetic tree based on sequences of the T3SS gene *rhcJ* was also constructed (Figure A2). The *rhcJ* genes of strains ORS3257, DOA1, DOA9, and SUTN9‐2 were obviously distinct from those in the non‐PB and PB clusters and the *B. elkanii* group. To more clearly understand the evolution of T3SSs among bradyrhizobial strains, the genomic arrangements of several T3SS clusters were determined using the program GenomeMatcher (Ohtsubo, Ikeda‐Ohtsubo, Nagata, & Tsuda, 2008) at the amino acid level (Figure 1). The annotated genome sequences of USDA110 (accession number BA000040), USDA61 (accession number FM162234.1), NGR234 (accession number NC_000914.2), and MAFF303099 (accession number NZ_CP016079.1) were obtained from Genome Assembly/Annotation Projects (NCBI database). The genome sequences of SUTN9‐2 (accession number LAXE00000000), DOA1 (accession number JXJM01000000), and DOA9 (accession number DF820426) were available in the DDBJ/GenBank/EMBL database. The genome sequence of ORS3257 was received from the MicroScope platform (Vallenet et al., 2016). The T3SS gene clusters were separated into three clusters based on their T3SS structural components (Figure 1a). The T3SS of bradyrhizobia displayed more notable differences compared with those of *S. fredii* NGR234 and *Mesorhizobium loti* MAFF303099. Region I of the bradyrhizobial T3SS cluster was similar to those found in all of the selected bradyrhizobial strains. The T3SS clusters of the Thai strains (SUTN9‐2, DOA1, and DOA9) and the Senegalese strain (ORS3257) were perfectly conserved. However, the T3SS gene organization in region II from the Thai strains and ORS3257 was partially different from that from USDA61 and USDA110. A distinct feature of the USDA110 T3SS cluster was the presence of several open reading frames (ORFs) that were absent in the other bradyrhizobial species. Furthermore, region II lacked *nopX* and no homologue was present in the USDA110 genome. The T3SS region III clusters of the bradyrhizobial strains were diverse. The T3Es *nopE1* and *nopE2* were detected only in USDA110, whereas *nopM* and *nopX* were absent in its genome (Figure 1b). Several putative T3Es could not be found in DOA9 and SUTN9‐2. It seems that the variation in the T3Es was higher than that in the T3SS structural core components. Based on the phylogenetic tree data and the gene arrangements, the T3SSs of the Thai strains (SUTN9‐2, DOA1, and DOA9) and ORS3257 may share the same origin. Thus, the bradyrhizobial strains SUTN9‐2, DOA1, DOA9, and ORS3257 evolved their T3SSs independently of the other bradyrhizobia because of biological and/or geological events. If this scenario is true, then it could be hypothesized that horizontal gene transfer (HGT) plays an important role in bradyrhizobial evolution. The T3E genes *nopP* and *nopL* were present in all of the bradyrhizobial strains, whereas homologues of these genes are not present in phytopathogenic bacteria (Ausmees et al., 2004; Bartsev, Boukli, Deakin, Staehelin, & Broughton, 2003; Bartsev et al., 2004). These data may also indicate that bradyrhizobia developed their T3Es independently of phytopathogenic bacteria. Therefore, these results may provide evidence confirming bradyrhizobial evolution.

**Figure A3:**
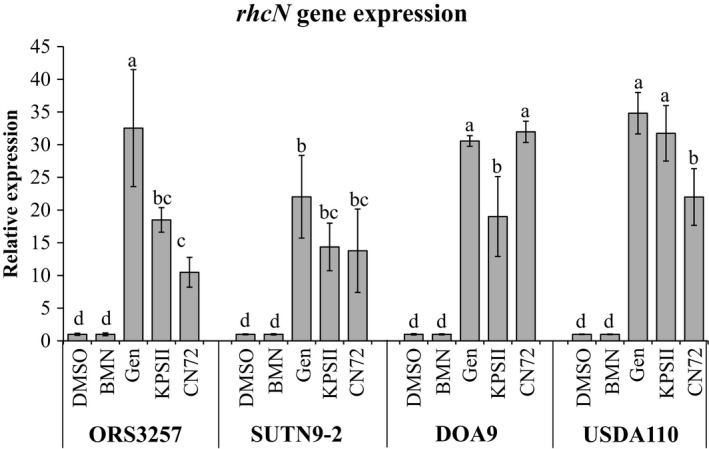
The relative expression of *rhcN* gene of *Bradyrhizobium* sp. STM6978 (STM6978), *Bradyrhizobium* sp. SUTN9‐2 (SUTN9‐2), *Bradyrhizobium* sp. DOA9 (DOA9), and *Bradyrhizobium diazoefficiens *USDA110 (USDA110) after induction with genistein (Gen) and mung bean (*Vigna radiata*) root exudates. The root exudates from mung beans were indicated as KPSII = V*. radiata* cv. KPSII, and CN72 = *V. radiata *cv. CN72. The DMSO and sterilized plant (BMN) medium were used as negative control. The level of expression was measured using qRT‐PCR. Values represent mean ± *SD* (*n* = 3). Within‐treatment means labeled with different letters are statistically different at *p* ≤ 0.05

Previous reports showed that *Vigna* are always found to establish symbiotic interactions with *Bradyrhizobium* spp. (Appunu, N'Zoue, Moulin, Depret, & Laguerre, 2009; Yokoyama et al., 2006; Zhang et al., 2008) and that bradyrhizobial strains have been isolated from various *V. radiata* ssp. in Thailand (Yokoyama et al., 2006). Furthermore, *V. radiata* has been continuously cultivated in Thailand; thus, it is a good plant model for understanding mutualistic properties of Thai bradyrhizobial strains. To obtain more information about the co‐evolution between *V. radiata *and bradyrhizobial T3SSs, the bradyrhizobial strains SUTN9‐2 (a native *V. radiata* symbiont in Thailand), DOA9 (a legume broad host range strain), ORS3257 (a native *V. unguiculata* symbiont in Senegal; Krasova‐Wade et al., 2003), and USDA110 (a *Glycine max* symbiont) were used in these experiments (Table A1). In our preliminary study of T3SS functions, the expression of *rhcN* was measured after induction with genistein (20 μM genistein dissolved in DMSO) and mung bean root exudate inductions (1/3 (v/v) of the root exudates). Primers for amplification are listed in Table A2. The effects of T3SS on *Bradyrhizobium*–*V. radiata* ssp. symbiosis could be separated into two groups (Figure A3). The *V. radiata* cv. KPSII (representing the incompatible group), in which the T3Es from all of the tested bradyrhizobial strains (except SUTN9‐2) strongly inhibited nodulation, and *V. radiata* cv. CN72 (representing the compatible group), in which it seems that the T3Es did not have negative effects on symbiosis (except the T3Es from ORS3257), were selected for root exudate preparation. The results of this experiment revealed that *rhcN* expression was activated by genistein and *V. radiata* root exudates in all of the tested bradyrhizobia 12 hr after induction (Figure A3). These data also raise the possibility that the T3SS machinery is formed during the early steps of the interaction between bradyrhizobial strains and these two *V. radiata* cultivars. Therefore, the T3SS is one of the factors that controls the *V. radiata* bradyrhizobia interaction at every step (Krause, Doerfel, & Göttfert, 2002).

**Figure 2:**
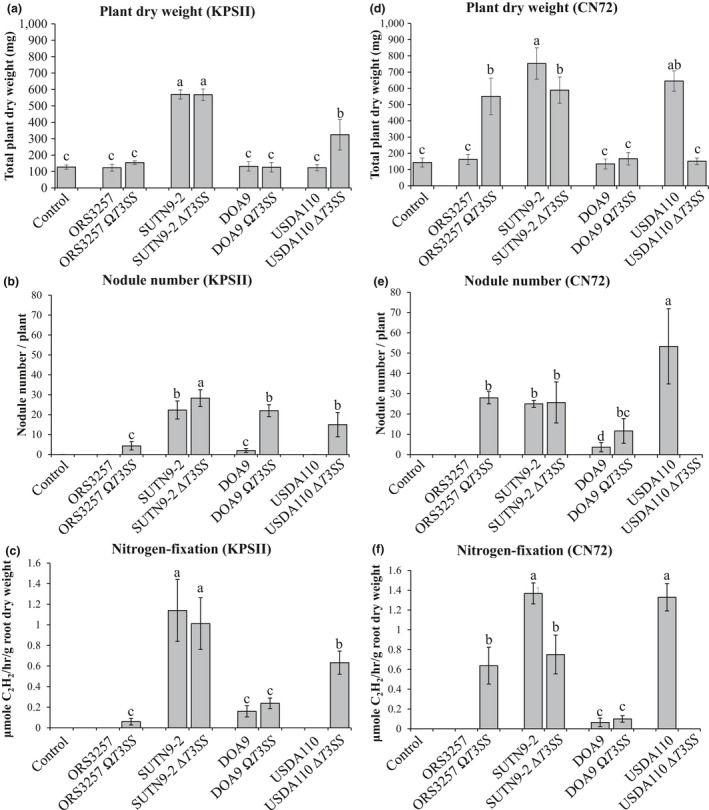
Nodulation and plant growth promotion by *Vigna radiata* cv. KPSII and *V. radiata* cv. CN72 inoculated with wild‐type (WT) and the three mutant TTSS strains. Total dry weights (a, and d), nodule number (b and e), and nitrogen fixation (c and f) are shown for the two different cultivars (abc, KPSII; def, CN72). Significance at *p* < 0.05 is indicated by the means and standard deviation bars (*n* = 3)

To examine whether the tested bradyrhizobial strains were *V. radiata* symbionts, they were inoculated into various *V. radiata* hosts (Table A3). The injectisome mutant strains ORS3257 (ORS3257 *ΩT3SS*: *rhcN *disruption; Okazaki et al., 2016), SUTN9‐2 (SUTN9‐2 *∆T3SS*: *rhcJ *deletion; Piromyou et al., 2015), DOA9 (DOA9 *ΩT3SS*: *rhcN* disruption) (Songwattana et al., 2017), and USDA110 (USDA110 *∆T3SS*: *nolB, rhcJ, nolU, and nolV *deletion; Krause et al., 2002) were used in this experiment (Table A1). It seems that the T3SSs of strains ORS3257, DOA9, and USDA110 showed negative effects on *V. radiata* (incompatible) symbiosis, but not on the *V. radiata* (compatible) group (Figure 2). Interestingly, the ORS3257 wild‐type strain could not form nodules in *V*. *radiata* ssp., whereas the ORS3257 *ΩT3SS* strain could establish symbiotic nodules with all of the tested *V*. *radiata* ssp. cultivars. The symbiotic characteristics of the wild‐type and T3SS mutants of the Thai strains (SUTN9‐2 and DOA9), ORS3257, and USDA110 were also evaluated with *V. radiate *cv. KPSII and *V. radiata* cv. CN72. The promotion of *V. radiata* cv. KPSII growth by the SUTN9‐2 wild‐type strain was significantly higher compared to all of the other treatments. On the other hand, the wild‐type strains ORS3257, DOA9, and USDA110 could not significantly promote *V. radiata* cv. KPSII growth compared with the uninoculated control (Figure 2a). The nodule number and nitrogen fixation properties of SUTN9‐2 were significantly higher than those plants inoculated with DOA9, while ORS3257 and USDA110 could not form symbiotic nodules (Figure 2b and 2c). For *V. radiata* cv. CN72, USDA110 could produce up to 50 nodules per plant at 28 days after inoculation (dai; Figure 2e). The SUTN9‐2 strain could also form symbiotic nodules, but with a significantly lower number compared with plants inoculated with USDA110. In contrast, nodulation of *V. radiata* cv. CN72 could not be detected after inoculated with ORS3257, while DOA9 could form a small number of nodules (approximately 5 nodules per plant). The nitrogen fixation of DOA9 was also lower than that resulting from SUTN9‐2 and USDA110 inoculations (Figure 2f). Based on the results with the T3SS mutants, it seems that bradyrhizobial T3SSs were less important for *V. radiata* bradyrhizobia symbiosis (except for *V. radiata* CN72). However, all of the tested bradyrhizobial strains still maintained the T3SS injectisome in their genomes, while they evolved their T3Es independently of other bradyrhizobia. Perhaps the bradyrhizobial T3SS was important for symbiosis with other legumes (Okazaki et al., 2013; Viprey et al., 1998). Therefore, some T3Es from ORS3257 and DOA9 showed negative effects on nodulation efficiency. Furthermore, the nodulation results implied that the mung bean cultivar is one of the factors that controls the compatibility of *V. radiata* bradyrhizobia symbiosis. This phenomenon reflects the bradyrhizobial host specificity of Thai bradyrhizobial strains for Thai *V. radiata* ssp. cultivars (Figure 2 & Table A3). In addition, the current symbiotic state of SUTN9‐2 has perfectly adapted to every tested *V. radiata* ssp. cultivar, whereas DOA9 cannot form effective nodules in any of the tested cultivars.

**Figure A4:**
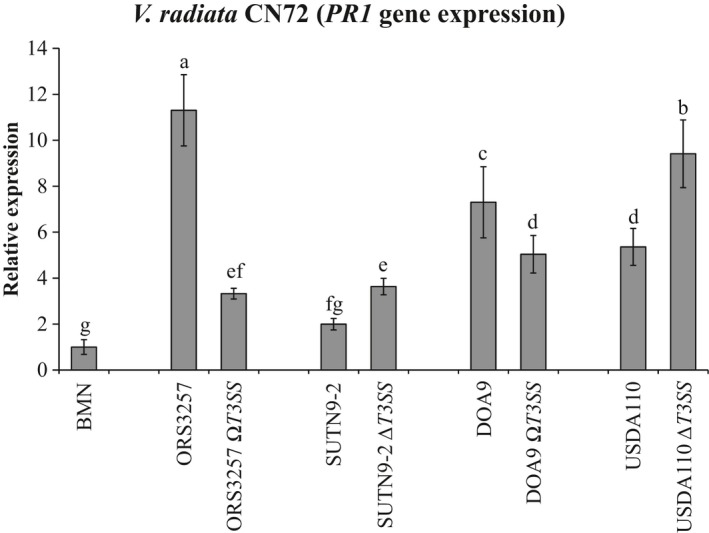
The relative expression of *PR1* gene of *Vigna radiata* CN72 at 2 dai with *Bradyrhizobium* sp. ORS3257 (ORS3257), *Bradyrhizobium* sp. ORS3257 T3SS mutant strain (ORS3257 *ΩT3SS*), *Bradyrhizobium* sp. SUTN9‐2 (SUTN9‐2), *Bradyrhizobium* sp. SUTN9‐2 T3SS mutant strain (SUTN9‐2 *∆T3SS*), *Bradyrhizobium* sp. DOA9 (DOA9), *Bradyrhizobium* sp. DOA9 T3SS mutant strain (DOA9 *ΩT3SS*), *Bradyrhizobium diazoefficiens *USDA110 (USDA110), and *Bradyrhizobium* sp. USDA110 T3SS mutant strain (USDA110 *∆T3SS*). The sterilized plant (BMN) medium was used as negative control. The level of expression was measured using qRT‐PCR. Values represent mean ± *SD* (*n* = 3). Within‐treatment means labeled with different letters are statistically different at *p* ≤ 0.05

**Figure A5:**
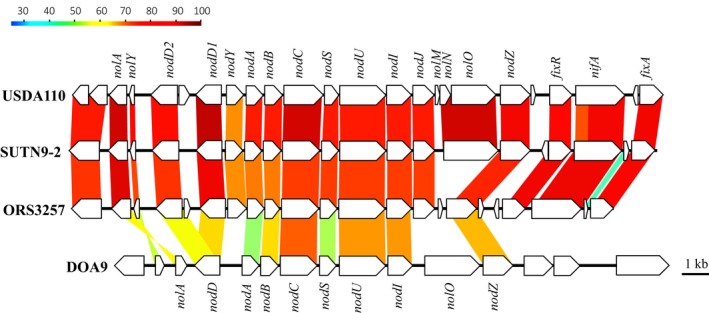
Comparison of the gene organization of nodulation (*nod*) cluster in strain, *Bradyrhizobium diazoefficiens* USDA110, *Bradyrhizobium* sp. SUTN9‐2, *Bradyrhizobium* sp. ORS3257, and *Bradyrhizobium* sp. DOA9. Orientations and sizes of the predicted ORFs are depicted by open arrows. Colored strips represent the conserved gene regions between the compared strains, and the color indicates the similarity percentage

The T3SS mutation experiments showed that the T3SSs had cultivar‐specific effects on nodulation properties. Wild‐type ORS3257 cannot form nodules with *V. radiata *cv. KPSII or cv. CN72, but both *V. radiata* cultivars readily formed nodules after ORS3257 *ΩT3SS* inoculation (Figure 2b,e). Similarly, DOA9 *ΩT3SS* had improved nodulation in *V. radiata *cv. KPSII and cv. CN72. Thus, the T3SSs of ORS3257 and DOA9 displayed negative effects on *V. radiata *cv. KPSII and cv. CN72 nodulation. In the case of USDA110, the T3SS showed negative effects on *V. radiata *cv. KPSII but not on cv. CN72 (positive effect). The T3SS of SUTN9‐2 had no effect on nodulation in both mung bean cultivars*.* Our results revealed that features of the T3SS seem to be important determinants of root nodule formation in *V. radiata*. To explore the T3SS‐dependent regulation of *V. radiata* defense mechanisms, we compared the expression of the *Pathogenesis‐Related 1* (*PR1*) gene in *V. radiata* CN72 roots inoculated with the wild‐type and T3SS mutant strains at 2 dai (Figure A4). The *PR1* gene was expressed at a very low level in the uninoculated control; however, the expression level was significantly enhanced by inoculation with most of the bradyrhizobial strains except SUTN9‐2. In *V. radiata* CN72 roots, the *PR1* gene expression levels following inoculation with wild‐type ORS3257 and DOA9 strains were significantly higher than those after inoculation with the T3SS mutant strains (ORS3257 *ΩT3SS *and DOA9 *ΩT3SS*). On the other hand, *PR1* expression was significantly enhanced after inoculation with the T3SS mutant strains (SUTN9‐2 *∆T3SS* and USDA110 *∆T3SS*) compared with the levels after inoculation with the wild‐type strains (SUTN9‐2 and USDA110). These results indicated that the T3Es from ORS3257 and DOA9 mediated defense signaling during the early stage of nodulation, whereas the T3Es from SUTN9‐2 and USDA110 could reduce *PR1* expression in the associated *V. radiata* CN72 roots. Therefore, *PR1* is one of the *V. radiata* CN72 mechanisms that controls nodulation efficiency. Some reports revealed that nodule formation generally begins with the exchange of chemical signals between the bradyrhizobia and compatible *V*. *radiata* roots. Each bradyrhizobial strain is adapted to recognize the flavonoids secreted by its compatible host. Recognition of the flavonoids by the symbiont results in the secretion of Nod factor, which is then tuned based on recognition by the *V*. *radiata* host (Geurts & Bisseling, 2002; Göttfert, Grob, & Hennecke, 1990; Long, 1996). Based on these phenomena, we hypothesized that Nod factor is the main factor required for *V. radiata* ssp. symbiosis; however, the T3SS is also important for host specificity. To better understand the relationship between the T3SS and the nodulation (*nod*) genes in *V. radiata* ssp. symbiosis, the Nod clusters were preliminary identified at the amino acid level using the GenomeMatcher program (Ohtsubo et al., 2008; Figure A5). The Nod clusters of strains SUTN9‐2 and ORS3257 were similar to that found in USDA110, whereas the Nod cluster of DOA9 was diverse. In addition, the nodulation‐mutant strains SUTN9‐2 (SUTN9‐2 Ω*nodABC*) and DOA9 (DOA9 Ω*nodB*) lost their symbiotic properties with *V. radiata* ssp. (data not show). It seems that *V. radiata* ssp. were promiscuous plants with diverse Nod factors. These results were consistent with previous reports that *V. radiata* is one of the most promiscuous plants (Yokoyama et al., 2006; Zhang et al., 2008). However, nodulation efficiency was also co‐regulated by the T3SSs (Figure 2 and Table A3; Krause et al., 2002; Krishnan et al., 2003). Therefore, it has been clearly demonstrated that effector‐triggered immunity (ETI) can determine genotype‐specific nodulation and that abolition of some T3Es can affect nodule formation in different ways, ranging from no effect to inducing reductions or increases in nodulation efficiency (Bellato, Krishnan, Cubo, Temprano, & Pueppke, 1997; Lorio, Kim, & Krishnan, 2004; Meinhardt, Krishnan, Balatti, & Pueppke, 1993; Viprey et al., 1998). However, DOA9 could also form ineffective nodules with every *V. radiata* cultivar tested. Perhaps DOA9 is an evolutionary intermediate between *V. radiata* symbiotic and non‐*V. radiata* symbiotic strains, as suggested by the difference in some T3Es and type III secretion promoters (*tts* boxes) in DOA9 compared with the compatible strain SUTN9‐2 (Table A4). In addition, the SUTN9‐2 T3Es are more likely to avoid recognition and/or suppression by the *V. radiata* defense mechanism. The T3Es of bradyrhizobial strains display mutualistic co‐evolution with *V. radiata* ssp. Our data support the idea that mutualism can result in host specificity and that bradyrhizobial mutualists may be under pressure from the host that limits diversification. This model could explain why the T3Es and *tts* boxes are diverse among bradyrhizobial species. Therefore, this work will provide a very logical transition into further study of how variations in the specific T3E content contribute to immune recognition.

**Figure 3:**
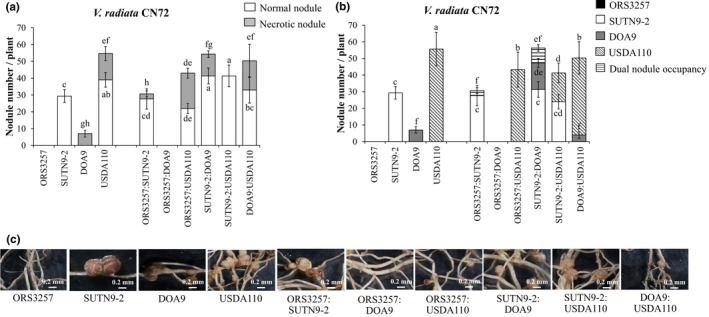
(a) Nodule phenotype (normal nodules or necrotic nodules), (b) nodule number, and (c) nodule phenotype of *Vigna radiata* cv. CN72 inoculated with single or with co‐inoculation of *Bradyrhizobium* sp. ORS3257, *Bradyrhizobium* sp. SUTN9‐2, *Bradyrhizobium* sp. DOA9, and *B. diazoefficiens* USDA110. Significance at *p* < 0.05 is indicated by means and standard deviation bars (*n* = 3)

**Figure 4:**
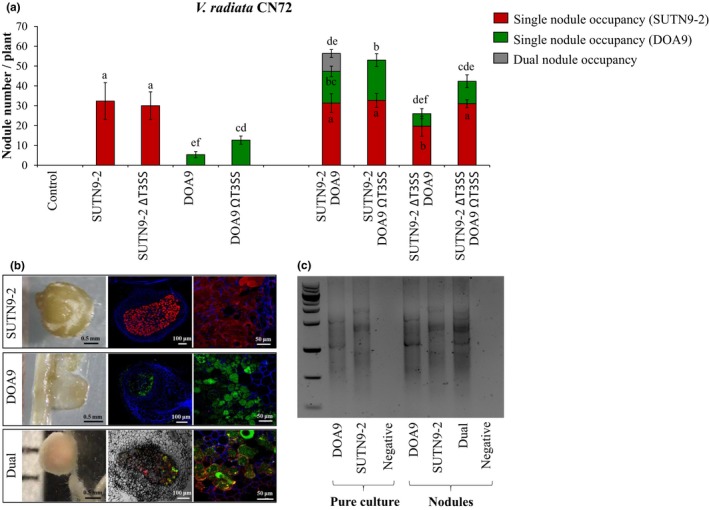
Nodule occupancy: (a) Nodule number of *Vigna radiata* cv. CN72 inoculated with *Bradyrhizobium* sp. SUTN9‐2 (SUTN9–2), *Bradyrhizobium* sp. SUTN9‐2 T3SS mutant strain (SUTN9‐2 *∆T3SS*), *Bradyrhizobium* sp. DOA9 (DOA9), and *Bradyrhizobium* sp. DOA9 T3SS mutant strain (DOA9 *ΩT3SS*). Significance at *p* < 0.05 is indicated by means and standard deviation bars (*n* = 3). (b) Nodule phenotype after co‐inoculation with SUTN9‐2 (SUTN9‐2 DsRed reporter gene‐tagged strain) and DOA9 (DOA9 gfp reporter gene‐tagged strain). (c) BOXA1R experiment: the nodule occupancy was identified using BOXA1R‐PCR

**Figure A6:**
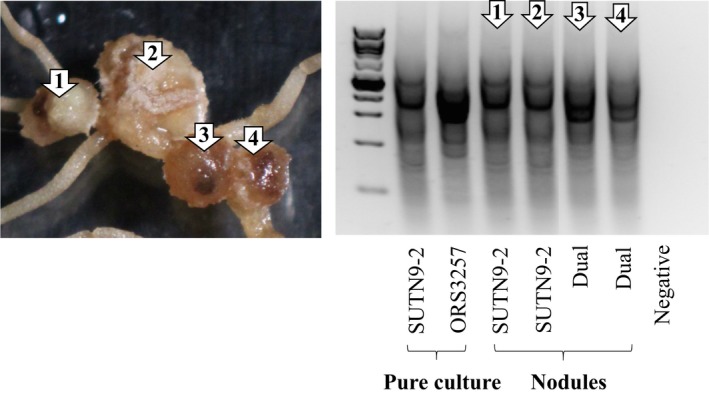
Nodule occupancy: Nodule phenotype of *Vigna radiata* cv. CN72 co‐inoculated with *Bradyrhizobium* sp. SUTN9‐2 and *Bradyrhizobium* sp. ORS3257. The DNA of bradyrhizobial strains (inside *V. radiata* nodules) was extracted, and then, the nodule occupancy was indicated by BOXA1R‐PCR. The number inside the arrow indicated the treatment (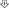 = BOXA1R‐PCR pattern 1 was carried out from *V. radiata* cv. CN72 nodule number 1)

Since SUTN9‐2 could nodulate all tested *V. radiata* cultivars, its T3Es seem to have effects on *PR1* expression. This property might be linked to its nodulation competition. However, the function of T3SSs in enhancing competition is still a mystery. To assess the competition of nodulation among bradyrhizobial strains, the nodule number and nodule occupancy of each pair, with cross‐inoculation with the same amount of living cells (10^6^ CFU/ml), were carried out using Leonard's jar experiments (Figure 3). Since *V. radiata* cv. CN72 was more promiscuous than cv. KPSII (Figure 2), it was selected for this experiment. Single inoculation with ORS3257 did not lead to the formation of symbiotic nodules in *V. radiata* cv. CN72, whereas nodulation without necrotic symptoms was detected after single inoculation with SUTN9‐2. Ineffective nodules in *V. radiata* CN72 were mostly found after DOA9 inoculation (Figure 3a,c). The strain USDA110 could form approximately 50 symbiotic nodules per plant, but senescent nodules (approximately 15 nodules per plant) were also detected. In the co‐inoculation experiment, necrotic nodules were found in each double inoculation, except for the SUTN9‐2:USDA110 inoculation (Figure 3a). ORS3257:USDA110 and DOA9:USDA110 co‐inoculations showed no significant changes in the numbers of necrotic nodules compared with USDA110 single and co‐inoculation treatments. To determine the nodule occupancy, bacterial genomic DNA was directly extracted from the surfaces of sterilized nodules. Next, the bradyrhizobia inside the nodules were identified using BOXAIR1‐PCR (Figure A6 and Figure 4c; Versalovic, Schneider, Bruijn, & Lupski, 1994). The nodule occupancy of SUTN9‐2 was not significantly different than resulting from single inoculation with SUTN9‐2 or from any of the co‐inoculations (Figure 3b). In contrast, the nodulation efficiency of DOA9 was entirely lost when it was co‐inoculated with ORS3257. The nodule number derived from USDA110 was also significantly reduced in the ORS3257:USDA110 co‐inoculation experiment. One possibility is that ORS3257 secreted some effector proteins that triggered the plant immunity and, consequently, the DOA9 and USDA110 strains also lost some of their capacity to form symbiotic nodules with *V. radiata* cv. CN72. On the other hand, the nodule number derived from SUTN9‐2 was not reduced after co‐inoculation with ORS3257. These results imply that SUTN9‐2 could ignore the plant immune response stimulated by ORS3557 and/or that SUTN9‐2 developed detoxification systems to overcome the plant defense mechanisms. Interestingly, dual occupancies were only found in the ORS3257:SUTN9‐2 and SUTN9‐2:DOA9 co‐inoculation experiments. This observation suggests that some ORS3257 or DOA9 cells could infect the same nodules with SUTN9‐2. Moreover, the singly inoculated ORS3257 could not form symbiotic nodules with *V. radiata* cv. CN72. Perhaps most of the nodules showing necrotic symptoms (as in the ORS3257:SUTN9‐2 co‐inoculation experiment) likely occurred due to the presence of the incompatible ORS3257 strain in the dual nodules. Based on these scenarios, it seems that SUTN9‐2 may limit the *V. radiata* cv. CN72 immune response, allowing ORS3257 to infect the plant host. However, *V. radiata* cv. CN72 still recognizes ORS3257 and the subsequent plant‐derived nodule senescence strategy eliminated the ORS3257 cheating cells. The *PR1* gene expression level might affect ORS3257 nodulation. However, the mechanisms used for ORS3257 infection (ORS3257:SUTN9‐2) are still unclear; therefore, ORS3257 infection processes will further be explored.

**Figure A7:**
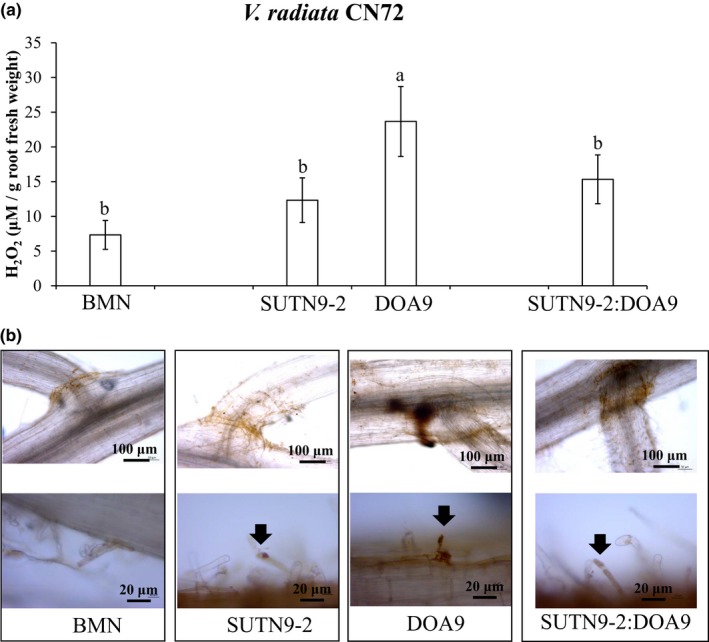
Quantitative and qualitative determination of H_2_O_2_ in *Vigna radiata* CN72 roots at 2 dai. *V. radiata* CN72 was inoculated with *Bradyrhizobium* sp. SUTN9‐2 (SUTN9‐2), *Bradyrhizobium* sp. DOA9 (DOA9), and co‐inoculation (SUTN9‐2:DOA9). The BMN was uninoculated treatment. (a) The accumulation of H_2_O_2_ was quantified in *V. radiata* CN72 roots using standard H_2_O_2 _concentration to calibrate data during the optical density (OD) measurements. (b) Roots were stained with 3,3′‐diaminobenzidine (DAB) solution (1 μg/ml DAB in 50 mM Tris‐acetate buffer, pH 5.0) for 8 hr. H_2_O_2_ levels correlate with the intensity of the brown color

To more clearly understand the function of the T3SS in SUTN9‐2 nodulation, the competition for nodule formation (cross‐inoculation) between wild‐type and T3SS mutant strains was explored in *V. radiata* cv. CN72 (Figure 4). After single inoculation, SUTN9‐2 and SUTN9‐2 *∆T3SS* could perform symbiotic nodules, whereas senescence nodules could be detected when *V. radiata* cv. CN72 was inoculated with DOA9 (Figures 3 and 4). The DOA9 *ΩT3SS* strain could form significantly more nodules compared to DOA9. It seems that the T3Es of DOA9 could suppress *V. radiata* cv. CN72 nodulation. The nodule number derived from SUTN9‐2 was not significantly different compared to that resulting from co‐inoculation (SUTN9‐2 with DOA9 and SUTN9‐2 with DOA9 *ΩT3SS*). In contrast, the number of nodules derived from SUTN9‐2 *∆T3SS *was drastically reduced when it was co‐inoculated with DOA9 (SUTN9‐2 *∆T3SS *with DOA9). These results indicated that the T3Es of DOA9 can trigger a plant immune response that can reduce the nodulation efficiency of the SUTN9‐2 *∆T3SS *strain. Moreover, symbiotic nodules derived from SUTN9‐2 *∆T3SS* were still detected when it was co‐inoculated with DOA9 (SUTN9‐2 *∆T3SS *with DOA9). This result implied that SUTN9–2 may use some other mechanism (not only its T3SS) to reduce plant immunity. Some T3Es presumably act similar to those of plant pathogens to suppress the plant immune response to promote the infection process. Other evidence also supports a role of H_2_O_2_ in bradyrhizobial infection, nodule development, and nodule senescence (Montiel, Arthikala, Cárdenas, & Quinto, 2016). We used 3,3′‐diaminobenzidine (DAB) staining to compare H_2_O_2_ accumulation in the roots of *V. radiata* cv. CN72 inoculated with single bradyrhizobial strains (SUTN9–2 or DOA9) and co‐inoculation (SUTN9‐2:DOA9) (Figure A7; Jaemsaeng, Jantasuriyarat, & Thamchaipenet, 2018). The quantitative H_2_O_2_ production was also measured in *V. radiata* CN72 roots using optical density (OD) measurement (compared with standard H_2_O_2_; Yasuda et al., 2016). The quantitative measurements of H_2_O_2_ showed that DOA9 induced significantly higher H_2_O_2_ accumulation compared with the uninoculated (BMN) control (Figure A7a), whereas the H_2_O_2_ accumulation levels after SUTN9‐2 inoculation and co‐inoculation (SUTN9‐2:DOA9) were not significantly different compared with the uninoculated control. The formation of a brown color reflects the level of H_2_O_2_ accumulation in the *V. radiata* cv. CN72 root (Figure A7b). H_2_O_2_ production was clearly found at the junction of the lateral roots and root hair zone after each bradyrhizobial inoculation. The roots were strongly stained (brown color) after inoculation with DOA9, whereas the brown color was drastically reduced after inoculation with SUTN9‐2. After co‐inoculation (SUTN9‐2:DOA9), the brown color seems paler than that caused by single DOA9 inoculation. This result indicated that DOA9 strongly induced H_2_O_2_ accumulation during the early stage of infection. However, H_2_O_2_ accumulation was also detected following SUTN9‐2 inoculation; therefore, the defense response was triggered transiently even during the compatible *V. radiata* bradyrhizobia interactions. In addition, SUTN9‐2 could suppress H_2_O_2_ production following co‐inoculation. Based on these results, we could confirm that SUTN9‐2 evolved its T3Es (signaling) to interact with *V. radiata* ssp. receptors and that these interactions likely weaken the plant immunity; therefore, some DOA9 cells take this opportunity to form *V. radiata* cv. CN72 root nodules (dual nodules; Figure 4 b,c). Consequently, it seems that SUTN9‐2 is the best‐adapted strain for *V. radiata* symbiosis. However, the symbiotic mechanisms of SUTN9‐2 are still partially unclear; therefore, the symbiotic relationships between SUTN9‐2 and *V. radiata* will further be explored.

**Figure 5:**
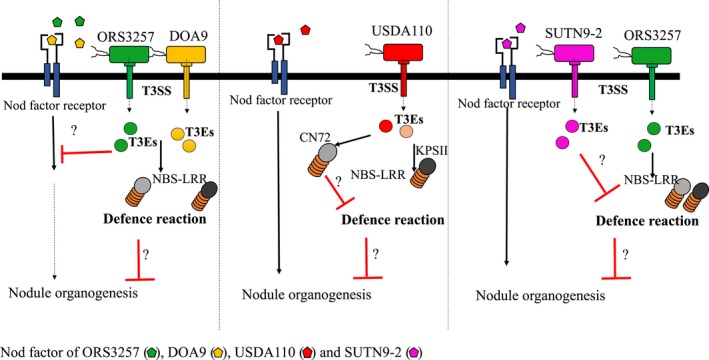
The model of mutualistic partnerships between *Vigna radiata* and their symbiont

We assume that successful establishment of *V. radiata–*bradyrhizobia symbiosis depends on how the bradyrhizobia have adapted to the special conditions on and in the *V. radiata* ssp. roots. Perhaps the T3Es from ORS3257 and DOA9 are directly bound by plant receptor CC‐nucleotide‐binding sites (NBS‐LRRs) inside the plant cells (Flor, 1971). In this situation, NBS‐LRRs strongly induce the *V. radiata* ssp. defense response, which ultimately blocks nodule formation. On the other hand, SUTN9‐2 could ignore the plant immune response and/or developed detoxification systems to overcome the effects of the plant defense mechanisms on nodule development. Nevertheless, the T3Es from USDA110 showed negative and positive effects on *V. radiata* ssp. symbiosis (Figure 5). Therefore, the host legumes and/or the environmental conditions are the main selective forces that drive the evolution of genes encoding functions involved in the symbiotic relationships of the microsymbionts. The mutualistic partnerships between *V. radiata* and their symbionts showed co‐evolution between SUTN9‐2 and *V. radiata*; thus, their mutualism may lead to selection of the most adapted combination of T3SS and symbiotic bradyrhizobial genotypes.

## CONFLICT OF INTEREST

The authors declare that there is no conflict of interest.

## AUTHORS CONTRIBUTION

P. P. and N. T. conceived and designed the experiment. P. P., P. S., and K. T. performed the experiment. P. P., and P. S analyzed the data.E. G., M. G., and N. T. provided bacteria used in this experiment.P. A. T. *provided mung bean* used in this experiment.P. P., P. T., N. B., and N. T. contributed to the critical discussion about the results. P. P., and N. T. wrote the manuscript. All authors read and approved the final manuscript.

## ETHICAL APPROVAL

This article does not contain any studies with humans or animals performed by any of the authors.

## Data Availability

The DNA sequences of the assembled genomes are available at NCBI under the BioProject accession: BA000040, FM162234.1, NC_000914.2, and NZ_CP016079.1. The genome sequences LAXE00000000, JXJM01000000, and DF820426 are available in the DDBJ/GenBank/EMBL database. The genome sequence of ORS3257 is received from the MicroScope platform Additional data sets generated in this work are provided in the Supporting Information Data S1.

## APPENDIX A

**Table A1:** Strains used in this study, sampling sites and relevant characteristics

Strain or Plasmid	Relevant characteristics and source of isolation	Source or reference
**Strains**		
***Bradyrhizobium*** **sp.**		
ORS3257	Native *Vigna unguiculata* symbiont in Senegal	(Krasova‐Wade et al., 2003)
SUTN9‐2	Isolated from paddy field using *Aeschynomene americana* as trap legume, native *v. radiata* symbiont in Thailand	(Noisangiam et al., 2012)
DOA9	Isolated from paddy field using *A. americana* as trap legume, legume broad host rang strain	(Noisangiam et al., 2012)
USDA110	*Glycine max* symbiont	(Greder et al., 1986)
ORS3257 *ΩT3SS*	*rhcN* disruption	(Okazaki et al., 2016)
SUTN9‐2 *∆T3SS*	*rhcJ* deletion	(Piromyou et al., 2015)
DOA9 *ΩT3SS*	*rhcN* disruption	(Songwattana et al., 2017)
USDA110 *∆T3SS*	*nolB, rhcJ, nolU, and nolV* deletion	(Krause et al., 2002)
SUTN9‐2DsRed	SUTN9‐2 marked with mTn*5*SS*DsRed* (pCAM120); *Sm* ^*r*^ *Sp* ^*r*^	(Piromyou et al., 2015)
DOA9GFP	DOA9 marked with *gfp* (pMG103‐npt2‐Sm‐npt2‐gfp); cloning vector harboring *gfp* gene under the control of the constitutive *npt2* promoter; *Sm* ^*r*^ *Sp* ^*r*^ *Km* ^*r*^	(Songwattana et al., 2017)

**Table A2:** Primers used in this study

Target gene	Primer name	Gene description	Primer sequence (5′→3′)	Description of design and reference
Bacterial genes				
**Endogenous housekeeping gene (16S rRNA)**				
16S rRNA	PBA**338**F PRUN518R	16S rRNA	ACTCCTACGGGAGGCAGCAG ATTACCGCGGCTGCTGG	(Songwattana et al., 2017)
**Type Three secretion System**				
*rhcN*	rhcN F‐ORS3257 rhcN R‐ORS3257	ATPase	TCGTTGTCGTTGAGACATCC AAAGACGGAAGGAGGAAAGC	Designed from *rhcN* of *Bradyrhizobium* sp. ORS3257 (The genome sequence was received from MicroScope platform.)
	rhcN F‐SUTN9‐2 rhcN R‐SUTN9‐2	ATPase	GCTCATAGGAGAGCGTGGAC CAGCGAATCCATCATCAGAA	Designed from *rhcN* of *Bradyrhizobium* sp. SUTN9‐2 **(LAXE00000000)**
	rhcN F‐DOA9 rhcN R‐DOA9	ATPase	CAGGTCGTTCTGATGATGGA ACCTTCGACAAGCACGGTAT	Designed from *rhcN* of *Bradyrhizobium* sp. DOA9 **(**DF820426**)**
	rhcN F‐USDA110 rhcN R‐USDA110	ATPase	TCCGATAGCGGAAGAATCAC CGGATCAGAGCCTTGTTTGT	Designed from *rhcN* of *Bradyrhizobium diazoefficiens* USDA110 (BA000040)
BOXAIR1	BOXA1R	BOXAIR1‐genomic patterns	CTACGGCAAGGCGACGCTGAC	(Versalovic et al., 1994)
Plant genes				
*PR1*	PR1‐F(1) PR1‐R(1)	Pathogenesis‐Related 1	TTGCACACCCGAGATGAATA TGACTGCAACCTTGAGCACT	Designed from *PR1* of *Vigna radiata* VC1973A
*Tubulin*	*VrTUB‐F VrTUB‐R*	Tubulin	CTTGACTGCATCTGCTATGTTCAG CCAGCTAATGCTCGGCATACTG	(Sairam et al., 2009)

**Table A3:** Nodulation test of bradyrhizobial strains

Leguminous plants	Nodulation test^a^							
	ORS3257	ORS3257 *ΩT3SS*	SUTN9‐2	SUTN9‐2 *∆T3SS*	DOA9	DOA9 *ΩT3SS*	USDA110	USDA110 *∆T3SS*
***Vigna radiata***								
**(incompatible)**								
‐cultivar KPS2	NO	YES (+)	YES (+)	YES (+)	YES (‐)	YES (‐)	NO	YES (+)
‐cultivar V4785	NO	YES (+)	YES (+)	YES (+)	NO	YES (‐)	NO	YES (+)
‐cultivar V4718	NO	YES (+)	YES (+)	YES (+)	NO	YES (‐)	NO	YES (+)
**(compatible)**								
‐cultivar SUT4	NO	YES (+)	YES (+)	YES (+)	YES (‐)	YES (‐)	YES (+)	YES (+)
‐cultivar CN72	NO	YES (+)	YES (+)	YES (+)	YES (‐)	YES (‐)	YES (+)	NO
***‐***cultivar V4758	NO	YES (+)	YES (+)	YES (+)	YES (‐)	YES (‐)	YES (+)	YES (+)

a Nodulation is indicated as follows: NO; no nodule detected, YES (‐); white nodules, Yes (+): reddish nodules.

**Table A4:** boxes localization of bradyrhizobial strains

**TTS BOX localization**	**Bradyrhizobia**			
	**ORS3257**	**SUTN9‐2**	**DOA9**	**USDA110**
**Inside TTSS cluster**				
*NopBA/nopM*	NO	YES	NO	NO
*nopB*	YES	YES	YES	YES
*rhcT*	YES	YES	YES	NO
*zinc protease*	NO	YES	NO	NO
*nopC*	YES	YES	YES	NO
*nopX*	YES	YES	YES	NO
*nopL*	YES	YES	YES	YES
**Outside TTSS cluster**				
*nopP*	YES	YES	YES	YES
*nopBW*	NO	NO	YES	NO
*nopM* (1)	YES	YES	NO	YES
*nopM* (2)	YES	YES	NO	NO
*nopM*1/2	YES	YES	NO	NO
*nopAF*	NO	YES	YES	NO
*nopAB*	YES	NO	NO	YES
*nopAE*	NO	NO	YES	NO
*Peptidase C48*	YES	YES	YES	NO
*Peptidase C58*	NO	YES	YES	NO
